# The feasibility of ultrasound-guided mini-percutaneous nephrolithotomy for ESWL-resistant lower calyx renal stones of up to two centimeters: a single center experience

**DOI:** 10.25122/jml-2023-0036

**Published:** 2023-04

**Authors:** Ali Hadi Sabhan, Ahmed Abdul Ameer Alwan

**Affiliations:** 1 Department of Surgery, College of Medicine, University of Al-Qadisiyah, Al-Diwaneyah, Iraq

**Keywords:** ESWL, mini-perc, PCNL, renal stone, ultrasound-guidance, COVID-19 – Coronavirus Disease 2019, CT – Computed Tomography, CXR – Chest X-ray, ECG – Electrocardiogram, ESWL – Extracorporeal Shock Wave Lithotripsy, Fr – French, IVP – Intravenous Pyelogram, KUB – Kidney Ureters and Bladder, PCNL – Percutaneous Nephrolithotomy, URS – Uretero-renoscopy

## Abstract

Lower pole renal stones present a significant challenge in urologic practice due to difficulty in accessing the calyx and eliminating fragments. Management options for these stones include watchful waiting for asymptomatic stones, extracorporeal shock wave lithotripsy (ESWL), ureterorenoscopy (URS), and percutaneous nephrolithotomy (PCNL). Mini-PCNL is a newer modification of conventional PCNL. The study aimed to assess the feasibility of mini-PCNL in treating lower pole renal stones equal to or less than 20mm that were not responsive to ESWL therapy. We included 42 patients (24 male and 18 female) with a mean age of 40±2.3 who underwent mini-PCNL at a single urology center between June 2020 and July 2022 and assessed operative and postoperative outcomes. The mean total operative time was 47±3.11 minutes, ranging from 40 to 60 minutes. The stone-free rate was 90%, and the overall complication rate was 26%, which included minor bleeding (5%), hematuria (7%), pain (12%), and fever (2%). The mean hospital stay was 80±3.34 hours (3-4 days). Our findings suggest that mini-PCNL is an effective treatment option for lower pole renal stones that are not responsive to ESWL therapy. The immediate stone-free rate was high, with minimum non-serious complications.

## INTRODUCTION

The primary challenge for patients with renal stones is not stone disintegration but rather the elimination of fragments. The goal of renal stone treatment is to relieve patients’ symptoms while preserving their renal function and eliminating the stone. Several factors influence the selection of effective therapy for calyceal calculi. These factors include the structure of the urinary system, the patient's overall health, and parameters connected to stones (size, site, and chemical components) [1]. Lower pole renal stones, which account for approximately 35% of all renal stones, are difficult to access and remove, making their treatment more complex [2].

At present, renal calculi can be treated using a variety of methods, such as shock wave lithotripsy or endoscopic stone removal via ureteroscopy or nephroscopy. Percutaneous nephrolithotomy (PCNL) is another option that involves the percutaneous removal of kidney stones and has been refined over time to minimize renal injury through mini-PCNL. The success rate depends on the stone burden and its architecture, the patient’s body, the structures of the urinary tract, and the surgeon's skills. These techniques also vary in anesthetic demands, level of invasiveness, stone extraction techniques, complications, and expenditures [3].

The treatment of intermediate-sized (≤20 mm) lower-pole stones is still argumentative. Extracorporeal shock wave lithotripsy (ESWL) is a minimally or non-invasive technique that does not require anesthesia and can be performed in multiple sessions with few assistants. However, the drawbacks are a lower stone-free rate, the need for additional procedures, and an increase in the rate of residual fragments when treating a high stone burden [4,5].

While PCNL has a higher morbidity rate, it may be preferred over ESWL as a first approach, with the option of salvage therapy with PCNL if the initial ESWL treatment fails [6]. According to current European Association of Urology guidelines, PCNL is the therapy of choice for larger renal stones (>20 mm) and lower calyceal stones ranging from 10 to 20 mm with adverse outcomes for ESWL [7].

The conventional PCNL procedure has evolved in terms of both tools and methodology, and it remains a less invasive treatment option today. With advancements in technology, the difficulties associated with this practice have decreased. These improvements include the manufacturing of smaller-size sheaths and the use of nephroscopy in mini-PCNL, as well as the utilization of sealing agents for the accessing tract, the application of regional anesthetics blocks, and eliminating the need for a nephrostomy catheter [8].

The newest smaller and portable ultrasound machines allow urologists to use them in the operating room for real-time imaging during stone targeting and removal. Ultrasonography-guided PCNL was initially mentioned in the 1970s [3] and has become increasingly popular in recent years, with numerous case studies demonstrating its practicality, safety, and effectiveness [9-11]. Studies have shown it is comparable to, and sometimes even superior to, fluoroscopic-guided PCNL regarding stone clearance [12,13], operative time [12], bleeding [12,13], and complications [12].

PCNL procedures are associated with significant morbidity, including bleeding, pain, and renal injury, often resulting from the use of larger instruments. To minimize these complications, a modified technique known as mini-PCNL involves using a smaller percutaneous tract (11-20 F) [14]. Helal et al. were the first to outline a method for mini-percutaneous nephrolithotomy in children [15], which has since been safe and successful in many studies, and can also be considered an option for adult patients [16-18].

Mini-PCNL is indicated for large renal stones (greater than 20 mm), ESWL-resistant stones, failed ureteroscopic clearance, and stones within the anatomically abnormal kidney, such as ectopic or transplanted kidney. Mini-PCNL may also be helpful for removing residual stone pieces after conventional PCNL [16,17,19]. The aim of our study was to assess the feasibility of mini-PCNL for patients with lower calyceal renal stones equal to or less than 20 mm who have not responded to initial treatment with ESWL.

## Material and Methods

### Patient selection

From June 2020 to July 2022, a total of 42 adult patients (24 male, 18 female) who failed initial treatment with ESWL for lower calyceal renal stones equal to or less than 20 mm in longest diameter were included in this prospective study. The study was approved by the ethical committee of the College of Medicine, Al-Qadisiyah University.

Exclusion criteria included pediatric age, patient unfit for anesthesia, bleeding tendency, presence of multiple calyces stones, stones larger than 20 mm, anatomically abnormal kidney like a horseshoe or ectopic kidney, and patient unwillingness for intervention.

Eligible patients had lower calyx renal stones or stones equal to or less than 20mm in longest diameter (for a single stone) or cumulative longest diameter (for more than one stone) that were not fragmented or failed to clear fragments following three or more consecutive standard sessions of ESWL.

Pre-operative evaluation included a complete medical history, performing a thorough physical examination, and conducting various investigations such as complete blood count, blood sugar, renal function test, virology screening (for hepatitis B and C virus and COVID-19), coagulation profile, urinalysis with or without culture/sensitivity testing and cardio-pulmonary assessment by electrocardiogram (ECG), echocardiogram (echo), chest x-ray (CXR), and pulmonary function test (PFT) when indicated. Stone characteristics (size, number, location, and density) were evaluated using abdominal ultrasound, kidney-ureter-bladder (KUB) X-ray, intravenous pyelography (IVP), and native computed tomography (CT) scan.

All patients achieved sterile urine, and those with infections underwent urine culture/sensitivity testing and received antibiotics based on the sensitivity results until clearance of the infection. Otherwise, surgery was postponed until the infection was eliminated.

### Surgical techniques

The surgical procedure and its possible complications were explained to the patients, and informed consent was obtained before surgery. All procedures were conducted at Al-Diwaneyah Teaching Hospital. General anesthesia was administered to all patients, and prophylactic antibiotics (Cephalosporin or Aminoglycoside) were administered parenterally at induction of anesthesia.

The patient was placed in the lithotomy position for cystoscopy, which allowed for the insertion of a double-J stent through the ureteral orifice of the targeted side. A Foley catheter was then fixed in place. The patient was then turned into the prone position for mini-PCNL. We chose to use these positions for all patients due to their widespread use and familiarity among the medical team.

The location of the kidney calyx, the site of the stone, and adjacent organs were assessed using ultrasound. Then, a 22-gauge Chiba needle was placed beside the ultrasound probe and used to puncture the lower calyx. To confirm the correct placement of the needle, the entire needle and its tip were inspected in the calyx near the stone. The guidewire was then coiled in the renal pelvis passing through the selected calyx, and urine efflux was observed from the external end of the needle. In cases where the access was incorrect, a second access was performed in the same way. Once the guidewire was placed, a small skin incision was made, and tract dilation was performed using 8F/14F coaxial dilators up to a size of 14 Fr. Then, a 14-Fr peel-away sheath was advanced into the tract, and a 12-Fr rigid nephroscope was passed for stone removal.

All patients included in the study had previously undergone ESWL before the mini-PCNL procedure. For patients with small fragmented stones, stone removal was performed using three-pronged grasping forceps. For those with non-fragmented stones, a pneumatic lithotripter was used for fragmentation, followed by extraction using forceps. A stone-free state was assessed intra-operatively by nephroscopy and ultrasound. In the absence of bleeding, perforation, peri-nephric collection, residual fragments, and multiple entrances to the calyx, the peel-away sheath was removed, and the skin incision was sutured by 2/0 silk. A nephrostomy tube (12 Fr.) was fixed into the kidney after removing the sheath.

### Follow-up

After the surgery, patients were closely monitored for their stone-free status using KUB, intravenous urography (IVU), or native CT scan. Stable patients were discharged from the hospital with antibiotic cover after removing the nephrostomy tube. Patients were advised to return to the hospital if any complications or abnormal conditions occurred. Otherwise, they were scheduled for a follow-up visit after 3 weeks for assessment and double-J stent removal, performed by cystoscopy under local anesthesia.

### Statistical analysis

In this prospective study, the patients' data, stone parameters, and surgical and postoperative results were all analyzed. The data were analyzed using the Statistical Package for the Social Sciences (SPSS) version 18. Descriptive statistics were used to present the data, with qualitative variables presented as frequencies and percentages, while quantitative variables were presented as mean and standard deviation.

## Results

Forty-two patients were enrolled in the study: 24 males (57%) and 18 females (43%), with a mean age of 40±2.3 (range from 27 to 55 years). Of these, 33 patients (79%) had a single lower calyx stone, while 9 patients (21%) had multiple lower calyx stones. The longest or cumulative longest diameter ranged from 11-20 mm, with a mean diameter of 16± 1.22. Twenty-eight patients (67%) had right-sided stones, and 14 (33%) had left-sided stones. Most stones (95%, n=40) appeared opaque on KUB, while the remaining 5% (n=2) were lucent. Stone density in CT-scan was not recorded due to cost considerations and the use of ultrasound in our technique.

All patients included in this study did not achieve successful fragmentation or clearance of their lower calyx renal stones following standard sessions of ESWL as initial therapy. Twenty-five patients (60%) underwent 3 consecutive standard sessions of ESWL, and 17 patients (40%) underwent more than 3 sessions (4-5 sessions). Twenty-nine patients (69%) had fragmented stones that failed to clear, while the remaining 13 patients (31%) had intact, non-fragmented stones. Table 1 presents the preoperative details of the patients and their stones.

**Table 1:** Patient and stone characteristics.

Variable			
**Age, years (mean±SD)**			7–55 (40±2.3)
**Gender, n (%)**	Male		24 (57)
	Female		18 (43)
**Stone characteristics**	Size, mm (mean±SD)		11–20 (16±1.22)
	Number of stones, n (%)	Single	33 (79)
		Multiple	9 (21)
	Side, n (%)	Right	28 (67)
		Left	14 (33)
	X-ray opacity, n (%)	Opaque	40 (95)
		Lucent	2 (5)
	Number of ESWL, n (%)	3 sessions	25 (60)
		>3 sessions	17 (40)
	Fragmented, n (%)	Yes	29 (69)
		No	13 (31)

During the mini-percutaneous nephrolithotomy procedure, access to the lower calyx was achieved with a single puncture in 32 patients (76%), while 10 patients (24%) required another puncture due to initial failure to provide appropriate access to the calyx. Four patients (9%) had a single residual fragment that was insignificant and not removed as it moved into a distant calyx. The operative time was estimated from the start of cystoscopy for double-J insertion until fixation of nephrostomy tube or closure of skin incision, and it ranged from 40-60 min with a mean 47±3.11.

A nephrostomy tube was inserted in 24 patients (57%), while the remaining 18 patients (43%) underwent skin closure without inserting a nephrostomy tube (tubeless). Stone-free status was achieved in 38 patients (90%) with complete removal of their stone burden without any residual fragments, as confirmed by intra-operative nephroscopy and ultrasound and later by further imaging studies. Table 2 presents the intraoperative results in detail.

**Table 2:** The intraoperative results of patients.

Variable		
**Operative time, min (mean±SD)**		40-60 (47±3.11)
**Stone free rate, n (%)**		38 (90)
**Residual fragment, n (%)**		4 (9)
**Number of punctures, n (%)**	Single	32 (76)
	Two	10 (24)
**Nephrostomy tube, n (%)**	Yes	24 (57)
	No	18 (43)

The study reported an overall complication rate of 26%, with non-serious complications occurring in 11 patients, including minor bleeding (n=2), hematuria (n=3), pain (n=5), and fever (n=1). No cases of visceral injury or sepsis were reported. The hospital stay duration ranged from 72-96 hours (3-4 days), with a mean of 80±3.34 hours. The nephrostomy tube was removed after 60-96 hours (mean 78±2.02 hours), and the double-J stent was removed after 21-28 days (mean 24±1.55 days). Table 3 shows the complications and postoperative data for the patients. It is noteworthy that no patient required additional intervention or conversion to conventional percutaneous nephrolithotomy.

**Table 3:** Complications and postoperative data.

Variable		
**Complications, n (%)**	Rate	11 (26)
	Bleeding	2 (5)
	Hematuria	3 (7)
	Pain	5 (12)
	Fever	1 (2)
	Visceral injury	0 (0)
	Sepsis	0 (0)
**Hospital stay, hours (mean±SD)**		72–96 (80±3.34)
**Time of catheter removal,**	PCN tube, hours (mean±SD)	60–96 (78±2.02)
	DJ stent, days (mean±SD)	21–28 (24±1.55)

PCN – Percutaneous Nephrostomy; DJ – Double-J.

## Discussion

The management of renal stones requires an optimal treatment modality that is minimally invasive and provides a high chance of complete stone removal in a single session. Extracorporeal shockwave lithotripsy is often the initial treatment modality, but alternative approaches may be necessary if ESWL fails [5]. All patients in this study underwent three or more sessions of ESWL as an initial treatment modality that failed to fragment or clear the stones from the lower calyx. The European Association of Urology (EAU) recommends PCNL or retrograde intrarenal surgery (RIRS) for the management of lower pole renal stones that are between 10-20 mm in length and have adverse factors for ESWL [7].

The mean stone size in our study was 16 mm, and most patients (79%) had a single stone. Karatag et al. [20] described their preliminary results with mini-PCNL for patients with medium-sized renal calculi. Their findings indicated that the mini-PCNL technique was safe and effective for kidney stones ranging in size from 10 to 20mm.

The mean operative time was 47 minutes, comparable to the findings of Knoll et al. [21] and Kirac et al. [22], who reported mean operative times of 48 and 53 minutes, respectively. This similarity in the duration of the procedure is likely due to the similar mean stone sizes in these studies, which were 18mm and 15mm, respectively, and 16mm in our study.

The stone-free rate in our study was 90%, which is comparable to previous reports, including 85.7% by Lee et al. [16], 96.5% by Hennessey et al. [17], and 91.9% by Kirac et al. [22].

Four patients (9%) had residual stone fragments, while 76% (n=32) had successful access to the lower calyx with a single puncture trial. Additionally, 43% (n=18) did not require a nephrostomy tube after surgery (tubeless mini-PCNL).

Altunrende et al. observed that 55% of patients with residual fragments would be clinically negligible or become free of stone at their follow-up, while 20% would become clinically significant, and 25% would require further intervention [23]. However, in our study, none of the patients with residual fragments required further intervention, as all fragments passed spontaneously during follow-up.

Most of our procedures (76%) were successful with a single puncture attempt, as we carefully selected patients with stones in a single lower calyx and thoroughly assessed their renal anatomy and stones using ultrasound.

Since the indications for tubeless PCNL are unclear, early tubeless PCNL was performed only in a subset of specific cases that included: stones less than 30 mm, non-complicated stones, absence of renal impairment, no multiple tract or perforation of collecting system, a short procedure time, complete stone evacuation, and lack of significant hemorrhage or hemodynamic instability [24]. Bellman et al. published the first series of tubeless PCNLs in 1997, which showed similar stone-free and complication rates in tubeless PCNL and ordinary PCNL patients with nephrostomy tubes [25].

The complication rate in our study was 26%, with grade 1 and 2 complications such as minor bleeding (5%), hematuria (7%), pain (12%), and fever (2%). These rates are comparable to those reported in recent studies on mini-PCNL by Abdelhafez et al. (23%), Long et al. (23.1%), Zeng et al. (25.9%), and Knoll et al. (28%) [21, 27-29]. We used a modified Clavien system for PCNL to categorize the complications in our study [26].

Pain was the most common complication in our study, occurring in the first 24 hours postoperatively. It was effectively managed with simple analgesia with paracetamol infusion with or without nonsteroidal anti-inflammatory drugs (NSAIDs). Hematuria was mild and resolved spontaneously, minor hemorrhage occurred in two patients, and hematuria and bleeding occurred in patients who underwent two trials of punctures. None of our patients required blood transfusion, and we observed that avoiding extensive angulation and multiple punctures was associated with minimum bleeding. One patient reported a febrile urinary tract infection and was treated with appropriate antibiotics without developing sepsis or serious events. None of our patients reported visceral or vascular injury.

Kumar et al. [30] reported that the most common complications in patients who underwent mini PCNL are pain, fever, frank hematuria, urinary tract infection, and urosepsis. Studies have shown that bleeding is directly related to the size of the sheath used during the procedure, with a smaller sheath resulting in reduced bleeding rates due to decreased exposure of the kidney parenchyma to injury [31,32].

A meta-analysis by Wan et al. [33] found that blood transfusion was less frequent in eight retrospective studies using a fixed-effects model analysis, and similar results were obtained in four non-retrospective studies. The blood transfusion rate was around 4% for patients treated by mini perc in another study [34].

Ultrasound guidance for access during mini-PCNL procedures allows for clear visualization of surrounding anatomy, which can help avoid bowel and visceral injury and enable accurate access to the targeted calyx. In contrast, fluoroscopic puncture through triangulation requires a more lateral angle of entry, which increases the risk of colonic trauma [35].

The mean hospital stay in our study was 80 hours (3.3 days), with a range of 72-96 hours (3-4 days). Pardalidis et al. [36] reported a mean hospital stay of 2.3 days, while Knoll et al. [21] and Mishra et al. [37] reported shorter hospital stays of 3.8 days and 3.2 days, respectively, in prospective comparative studies. Other studies have reported a hospital stay of approximately 74.4 hours [30,34].

## Conclusion

Mini-percutaneous nephrolithotomy has proven to be an effective and safe treatment option for lower calyx renal stones up to 20 mm that do not respond to ESWL therapy. It provides rapid stone clearance with high immediate stone-free rates and minimal non-serious complications in a single session. Ultrasound-guided access is a valuable technique that reduces patient and surgical personnel exposure to radiation and lowers the risk of inadvertent visceral injury. Further studies are needed to assess the long-term outcomes of this therapeutic option.
